# Supplementary material for: MetaRibo-Seq measures translation in microbiomes
Source: Nat Commun. 2020 Jun 29;11:3268. doi: 10.1038/s41467-020-17081-z (PMC7324362; doi:10.1038/s41467-020-17081-z)
Supplement: Supplementary file 10 — Supplementary Data 7 [file 41467_2020_17081_MOESM10_ESM.zip › File2/Confidence_VeryHigh_Taxonomy/345486_out.krona.html]

Javascript must be enabled to view this page.

members
magnitude
magnitudeUnassigned
count
unassigned
taxon
rank

345486\_out

40

superkingdom
40
2

phylum
40
1239

class
186801
40

186802
40
order

family
541000
40

genus
1263
1


SRS019496\_contig\_number\_3088
species
1262955
1

genus
39
292632

1262970
39
species

SRS011134\_contig\_number\_27040SRS011239\_contig\_number\_27082SRS011405\_contig\_number\_17973SRS012969\_contig\_number\_22136SRS015431\_contig\_number\_55290SRS016203\_contig\_number\_10045SRS018817\_contig\_number\_22651SRS018836\_contig\_number\_19898SRS019068\_contig\_number\_39591SRS020233\_contig\_number\_41619SRS020394\_contig\_number\_14308SRS022071\_contig\_number\_21195SRS022609\_contig\_number\_13450SRS023526\_contig\_number\_contig-100\_673.170477SRS024009\_contig\_number\_7068SRS024435\_contig\_number\_21635SRS048060\_contig\_number\_4127SRS049959\_contig\_number\_15168SRS049995\_contig\_number\_14792SRS052697\_contig\_number\_25380SRS064757\_contig\_number\_16858SRS065504\_contig\_number\_31517SRS075821\_contig\_number\_19391SRS075984\_contig\_number\_12568SRS076804\_contig\_number\_14733SRS077849\_contig\_number\_19363SRS078242\_contig\_number\_19838SRS098571\_contig\_number\_31577SRS1041039\_contig\_number\_21847SRS1041137\_contig\_number\_26882SRS104311\_contig\_number\_33998SRS104400\_contig\_number\_35948SRS142503\_contig\_number\_42128SRS142542\_contig\_number\_12983SRS143895\_contig\_number\_28559SRS144183\_contig\_number\_4576SRS147557\_contig\_number\_14040SRS971275\_contig\_number\_22866SRS971276\_contig\_number\_16907
